# Desmopressin After Kidney Biopsy: Are the Risks Worth it?

**DOI:** 10.1016/j.ekir.2025.06.058

**Published:** 2025-07-11

**Authors:** Taha Enes Cetin, Ozant Helvaci

**Affiliations:** 1 Department of Nephrology, Gazi University, Ankara, Turkey

To the Editor:

We read with great interest, the article by Prasad *et al.*^1^ titled “Randomized Double-Blind Placebo-Controlled Trial of Desmopressin for Post–Kidney Biopsy Bleeding.” The authors deserve recognition for conducting a well-designed randomized controlled trial on a key procedural issue in nephrology. The strengths include the rigorous methodology, nephrologist-performed biopsies, double-blind design, and the rare inclusion of von Willebrand factor and bleeding diathesis assessments.

That said, we believe that some aspects warrant critical reflection. The reported reduction in bleeding appears to stem predominantly from a decrease in minor complications, specifically transient macroscopic hematuria and self-limiting subcapsular hematomas. As acknowledged by the authors and widely recognized in the literature, such findings typically resolve spontaneously without intervention.^2^ In fact, the approximately 30% incidence of hematoma detected on postbiopsy ultrasound is consistent with previous reports in the field.^3^

The hyponatremia rate observed in the desmopressin group (∼29%) is markedly high and likely attributable to the relatively elevated intranasal dose used. This frequency exceeds that reported in most previous desmopressin studies^4^ and may pose significant risk in patients with fluid-sensitive conditions such as acute kidney injury, rapidly progressing glomerulonephritis, or heart failure, as well as in those with borderline hyponatremia at baseline.

Moreover, the desmopressin group exhibited a greater reduction in hemoglobin over time, despite the authors attributing this to hemodilution. In clinical practice, such decreases, especially in patients who are already anemic at baseline, may carry substantive risk. From our perspective, accepting complications such as hyponatremia, hemoglobin decline, headache, or even risk of volume overload in susceptible individuals in exchange for the prevention of mostly benign and transient bleeding events may not be a routinely justifiable approach.

Although we raise these concerns regarding clinical applicability and safety, the study nonetheless represents a valuable step forward in addressing procedural risk in nephrology. Given the rarity of investigator-initiated randomized controlled trials in the field, we commend the authors for their rigorous efforts and look forward to their future work with anticipation.

## References

[1] Prasad N., Meyyappan J., Yadav D.K. (2025). Randomized double-blind placebo-controlled trial of desmopressin for post–kidney biopsy bleeding. Kidney Int Rep.

[2] Poggio E.D., McClelland R.L., Blank K.N. (2020). Systematic review and meta-analysis of native kidney biopsy complications. Clin J Am Soc Nephrol.

[3] Feldmann Y., Böer K., Wolf G., Busch M. (2018). Complications and monitoring of percutaneous renal biopsy-a retrospective. Clin Nephrol.

[4] Lim C.C., Tan H.Z., Tan C.S., Healy H., Choo J., Franca Gois P.H. (2021). Desmopressin acetate to prevent bleeding in percutaneous kidney biopsy: a systematic review. Int Med J.

